# Sodium hyaluronate combined with rhEGF contributes to alleviate clinical symptoms and Inflammation in patients with Xerophthalmia after cataract surgery

**DOI:** 10.1186/s12886-022-02275-4

**Published:** 2022-02-07

**Authors:** Xuewu Gong, Hongbo Yao, Jing Wu

**Affiliations:** 1 Ophthalmology Department, The Second Affiliated Hospital of Qiqihar Medical University, No.37, Zhonghua West Road, Jianhua District, Qiqihar, 161006 Heilongjiang China; 2 School of Basic Medicine of Qiqihar Medical University, Qiqihar, 161006 Heilongjiang China

**Keywords:** Sodium hyaluronate, rhEGF, Xerophthalmia, Cataract surgery

## Abstract

**Background:**

To determine the effect of sodium hyaluronate combined with recombinant human epidermal growth factor (rhEGF) on clinical symptoms and inflammation in patients with newly diagnosed xerophthalmia after cataract surgery.

**Methods:**

A total of 106 patients who underwent cataract surgery and were newly diagnosed with xerophthalmia in our hospital between June 2018 and August 2019 were enrolled. Of these, 50 patients who were treated with sodium hyaluronate (0.1%) were assigned to the monotherapy group (MG) and the remaining 56 patients who were treated with sodium hyaluronate (0.1%) combined with rhEGF (20 μg/ml) were assigned to the combination group (CG). The 2 groups were compared based on ocular surface disease index (OSDI) score, break-up time (BUT), fluorescein corneal staining level, Schirmer I test (SI) level, clinical efficacy (disappearance of typical symptoms, including eyes drying, burning sensation, foreign body sensation, etc), and interleukin (IL)-1, IL-6, and tumor necrosis factor-α (TNF-α) levels. Spearman correlation analysis was conducted to analyze the relationship between IL-1, IL-6, TNF-α and clinical efficacy. In addition, receiver operating characteristic curves were drawn to analyze the predictive value of IL-1, IL-6, and TNF-α in efficacy on xerophthalmia. Results: After treatment, the CG showed reduced OSDI score compared with the MG. The CG showed increased BUT (s) and SI (mm) levels compared with MG. After treatment, the CG exhibited decreased levels of IL-1(ng/mL), IL-6 (ng/mL), and TNF-α (ng/mL) compared with the MG. Spearman correlation analysis revealed that IL-1, IL-6, and TNF-α were negatively correlated with clinical efficacy. The areas under the curves of IL-1, IL-6, and TNF-α were 0.801, 0.800, and 0.736 respectively.

**Conclusions:**

Sodium hyaluronate combined with rhEGF is helpful to alleviate clinical symptoms and inflammation in patients with xerophthalmia undergoing cataract surgery.

## Background

Cataract, which affects 46 million people worldwide, is the second leading cause of blindness and visual impairment in the global population [1, 2]. It is easy to treat, but remains the primary cause of vision impairment among the elderly in developed countries [3]. At present, cataract is primarily treated by surgery in clinical practice. With high safety and good curative effect, cataract surgery has been widely used clinically and has become the treatment of choice for cataract [4]. However, some patients experience xerophthalmia after surgery, which compromises the patient’s visual function and results in negative emotions, affecting the patient’s daily life and work [5, 6]. Therefore, intervention for patients with xerophthalmia after cataract surgery is crucial to improve the patient’s quality of life.

Patients often experience xerophthalmia and inflammation after undergoing cataract surgery. Xerophthalmia, as well as dye eye disease, occurs when tears aren’t able to provide adequate lubrication for eyes. Patients may experience a stinging, burning sensation, eye redness, blurred vision or eye fatigue. It is accompanied by tear film dysfunction and inflammation of the ocular surface [7]. Reasons for tear film dysfunction could include hormone changes, autoimmune disease, or allergic eye disease [8]. Aging, certain medical conditions and medicines as well as corneal nerve desensitivity also paly a critical role [9]. Meibomian gland dysfunction is the most common cause of evaporative dry eye [10]. Non-steroidal anti-inflammatory drugs are highly effective in reducing inflammation and the incidence of postoperative macular cystoid edema, but usually bring about adverse complications [11]. There are various treatments for xerophthalmia, including artificial tear, ointment, or gel, local secretagogue, anti-inflammatory therapy, and surgery. Low-level light therapy at 740 nm was effective in controlling the corneal conditions and the degree of inflammation in xerophthalmia [12]. Artificial tear is the most direct and minimally invasive treatment. Sodium hyaluronate, as an artificial tear, has been widely adopted in the treatment of xerophthalmia in recent decades [13]. Sodium hyaluronate has an obvious effect on ameliorating the symptoms of postoperative xerophthalmia, but its effect on alleviating inflammation is poor [14]. Epidermal growth factor is a single polypeptide chain composed of 53 amino acids that exists in the human body and can stimulate the proliferation of epithelial cells and the differentiation of epithelial tissues and promote skin regeneration and wound healing [15]. One study revealed that recombinant human epidermal growth factor (rhEGF) can be used in penetrating keratoplasty, refractive surgery, and the treatment of diabetic keratopathy and xerophthalmia, without resulting in adverse reactions [16]. A previous research also proved that hyaluronic acid sodium combined with rhEGF can show better therapeutic effects on the dry eye symptom than the eye drops alone following LASIK [17]. Therefore, we considered that sodium hyaluronate combined with rhEGF can promote the division and proliferation of corneal and conjunctival epithelial cells and cornea repair while alleviating the symptoms of xerophthalmia in patients after cataract surgery. Inflammatory response and oxidative damage are important in the pathogenesis of cataract [18], whereas the efficacy of sodium hyaluronate combined with rhEGF on the inflammatory response of patients remains unclear.

Therefore, this study used sodium hyaluronate combined with rhEGF to treat patients with xerophthalmia after cataract surgery and analyzed the effect of this combination on xerophthalmia symptoms and the inflammatory response of the patients, with the goal of providing valuable reference for clinical treatment.

## Materials and methods

### Clinical data of patients

A total of 106 patients with newly diagnosed xerophthalmia who underwent cataract surgery in our hospital between June 2018 and August 2019 were enrolled in this study. Of these, 50 patients who were treated with sodium hyaluronate were assigned to the monotherapy group (MG) and the remaining 56 patients who were treated with sodium hyaluronate combined with rhEGF were assigned to the combination group (CG). This study was approved by the Ethics Committee of the Second Affiliated Hospital of Qiqihar Medical University. The inclusion criteria included: 1) patients who were newly diagnosed with xerophthalmia after successful cataract surgery within 4 weeks, 2) patients meeting the diagnostic criteria of xerophthalmia [19], 3) patients with normal neurological function, 4) patients who cooperated with the treatment, 5) patients with complete clinical data, and 6) those who and whose family members signed informed consent forms after understanding the study. The exclusion criteria included: 1) patients who experienced congenital absence of the lacrimal gland or alacrimia congenita; 2) patients with severe liver or renal dysfunction; 3) patients who had taken systemic antihistamines, anticholinergic agents, or other ophthalmic drugs within the past month; and 4) patients who were pregnant or lactating.

### Sources of drugs and instruments

Sodium hyaluronate (H20150150; URSAPHARM Arzneimittel GmbH, Saarbrücken, Germany, 0.1%), rhEGF (20,020,016; Pavay Gene Pharmaceutical Co., Ltd., Guilin, China, 20 μg/ml), tumor necrosis factor-α (TNF-α) enzyme-linked immuno-sorbent assay (ELISA) kit (XF-HUMAN-1766; Xinfan Biotechnology Co., Ltd., Shanghai, China), interleukin (IL)-6 ELISA kit (XFH10605; Xinfan Biotechnology Co., Ltd.), IL-1 ELISA kit (ml058034; MLBIO Co., Ltd., Shanghai, China), and PHOMO automatic microplate reader (Zhongsheng Life Science Development Co., Ltd., Shanghai, China).

### Therapeutic regimen

Each patient in the MG was treated with sodium hyaluronate eyedrops at a dose of 1 drop at a time and 3 drops per day. Each patient in the CG was also treated with rhEGF eyedrops at a dose of 1 drop at a time and 3 drops per day. Both groups were treated continuously for 8 weeks.

### Determination methods

#### Break-up time

Fluorescein sodium solution was dropped into the conjunctival sac of each patient, and the patient was asked to blink several times and look at the front horizontally. Subsequently, the whole cornea of the patient was evaluated under the broad slit of cobalt-blue light of a slit lamp. The time from the last blinking to the appearing of the first black spot during consistent eye opening was the tear film break-up time (BUT). The BUT was measured 3 times and averaged. The normal BUT is between 10 and 45 s, and a BUT less than 10 s indicates instability of the tear film.

#### Fluorescein corneal staining

Fluorescein sodium solution (1%) was dropped into the conjunctival sac of each patient and was then evaluated with cobalt-blue light under a slit lamp microscope after the patient blinked. Non-pigmentation indicated normal corneal epithelium, and pigmentation indicated corneal epithelial defect. 0 points indicated non-pigmentation of the corneal epithelium; 1 point indicated that the pigmented area accounted for less than one-third of the total corneal area; 2 points indicated that the pigmented area accounts for more than one-third of the total corneal area but less than half; and 3 points indicated that the pigmented area accounted for more than half of the total corneal area.

#### Schirmer I test

No surface anesthetic was used on the ocular surface. The front end of a test paper was folded back at a marked place, and the front end of the test paper was gently placed at the outer third of the conjunctival surface of the lower eyelid of the tested person. During the test, the patient was instructed to avoid talking and rotating the eyeball so as not to affect the test results. After 5 min, the test paper was removed, and the length (mm) of test paper soaked by tear was recorded.

#### Elisa

The levels of inflammatory factors (IL-1, IL-6, and TNF-α) were quantified using ELISA as follows: tear (5 mL) was sampled from each patient in the 2 groups before and after treatment, let to stand at room temperature for 30 min, and centrifuged at 3000 g and 4 °C for 10 min to take the supernatant. The supernatant was stored in a refrigerator at − 80 °C for later analysis. A blank well, a standard well, and a well for samples to be tested were set, respectively. No enzyme-labeled reagent or sample was added into the blank well, and 100 ml samples to be determined and 100 mL standards were added into the well for samples to be determined and the standard well, respectively. The enzyme-labeled plate was covered with a film after the substance in each well was mixed well and incubated at 37 °C for 2 h. The liquid in each well was discarded, and they were shaken to dry before 100 μl of working fluid A was added. The plate was then covered with a film and incubated at 37 °C for 1 h. Subsequently, the liquid in each well was discarded, and the wells were shaken to dry and washed with washing solution 3 times. Then, 100 μl of working fluid B was added to each well, and the plate was covered with a film and incubated at 37 °C for 1 h. The liquid in each well was also discarded, and the wells were shaken to dry and washed with washing solution 3 times again before 90 μl of substrate solution was added. The wells were covered with a film and placed in the dark at 37 °C for development for 10–15 min. Finally, 50 μl of stopping solution was added to each well to terminate the reaction. The optical density of each well at 450 nm was determined, and the concentrations of IL-1, IL-6, and TNF-α were calculated.

### Outcome measures

The ocular surface disease index (OSDI) was adopted as the primary outcome measure to evaluate ocular symptoms, including 4 vision functions, 5 ocular discomfort symptoms, and 3 environmental trigger factors, with 0 to 4 points assigned for each item [20]. The final score was the sum of all scores multiplied by 25, then divided by the total number of evaluation items. A higher score indicated more serious symptoms of xerophthalmia. The OSDI scores of the 2 groups before and after treatment were compared, and the levels of BUT, fluorescein corneal staining (FL) level, and Schirmer I test (SI) before and after treatment were evaluated. In addition, the clinical efficacy on the 2 groups was analyzed. The total effective rate equaled the number of patients with effective treatment plus the number of patients with markedly effective treatment plus the number of cured patients, divided by the total number of patients, then multiplied by 100%. The clinical efficacy evaluation criteria are shown in Table 1. In addition, the levels of IL-1, IL-6, and TNF-α in the 2 groups before and after treatment were evaluated.

**Table 1:** Evaluation standards of clinical efficacy

Clinical Efficacy	Assessment Criteria
Cured	BUT > 10 s; FL: 0 points; SI > 10 mm; xerophthalmia symptom disappeared
Markedly effective	Two items of BUT, FL, and SI returned to normal, and the symptoms of xerophthalmia disappeared.
Effective	One item of BUT, FL, and SI returned to normal, and the symptoms of xerophthalmia disappeared partially.
Ineffective	Levels of BUT, FL, SI, and xerophthalmia symptoms did not improve or worsened.

Secondary outcome measures included Spearman correlation analysis, which was carried out to analyze the relationship between IL-1, IL-6, and TNF-α levels and clinical efficacy. According to the clinical efficacy on the patients, all patients were divided into 2 groups: a good efficacy group and a poor efficacy group. The levels of IL-1, IL-6, and TNF-α in the 2 groups before treatment were quantified, on which receiver operating characteristic (ROC) curves of them were drawn to evaluate the predictive value of IL-1, IL-6, and TNF-α in xerophthalmia after cataract surgery.

### Statistical analysis

In this study, the collected data were statistically analyzed using SPSS 20.0 (Cabit Information Technology Co., Ltd., Shanghai, China), and visualized into figures using Prism 7 (SOFTHEAD Software Technology Co., Ltd., Shenzhen, China). Enumeration data were expressed as rate (%), analyzed using the chi-square test, and expressed by χ^2^. Measurement data were expressed as the mean ± standard deviation, and were compared between groups using the independent-samples *t* test and compared within groups before and after treatment using the paired *t* test, both of which were expressed by *t*. In addition, the correlation between the levels of serum IL-1, IL-6, and TNF-α in patients and clinical efficacy was analyzed using Spearman correlation analysis. ROC curves were used to draw and analyze the predictive value of IL-1, IL-6, and TNF-α in the efficacy on xerophthalmia after cataract surgery. A *P* value of less than 0.05 indicated a significant difference.

## Results

### Comparison of baseline data

Comparison of baseline data between the 2 groups showed that no significant difference between the groups in terms of sex, age, body mass index, educational level, place of residence, smoking history, and drinking history was noted (all *P* > 0.05) (Table 2**)**.

**Table 2:** Comparison of baseline data between the 2 groups

Group	Monotherapy Group (*n* = 50)	Combination Group (*n* = 56)	χ^2^/*t*	*P* value
Sex				
Male	27 (54.00)	26 (46.43)	0.606	0.436
Female	23 (46.00)	30 (53.57)		
Age, y	66.8 ± 8.9	68.3 ± 9.1	0.856	0.394
BMI (kg/m^2^)	21.42 ± 2.15	22.03 ± 2.12	1.469	0.145
Education level				
<Junior diploma	22 (44.00)	27 (48.21)	0.189	0.664
≥Junior diploma	28 (56.00)	29 (51.79)		
Place of residence				
Urban area	26 (52.00)	25 (44.64)	0.573	0.449
Rural area	24 (48.00)	31 (55.36)		
Smoking history				
Yes	31 (62.00)	29 (51.79)	1.122	0.290
No	19 (38.00)	27 (48.21)		
Drinking history				
Yes	29 (58.00)	28 (50.00)	0.680	0.410
No	21 (42.00)	28 (50.00)		

*BMI* Body mass index

### Comparison of OSDI score

**Fig. 1:**
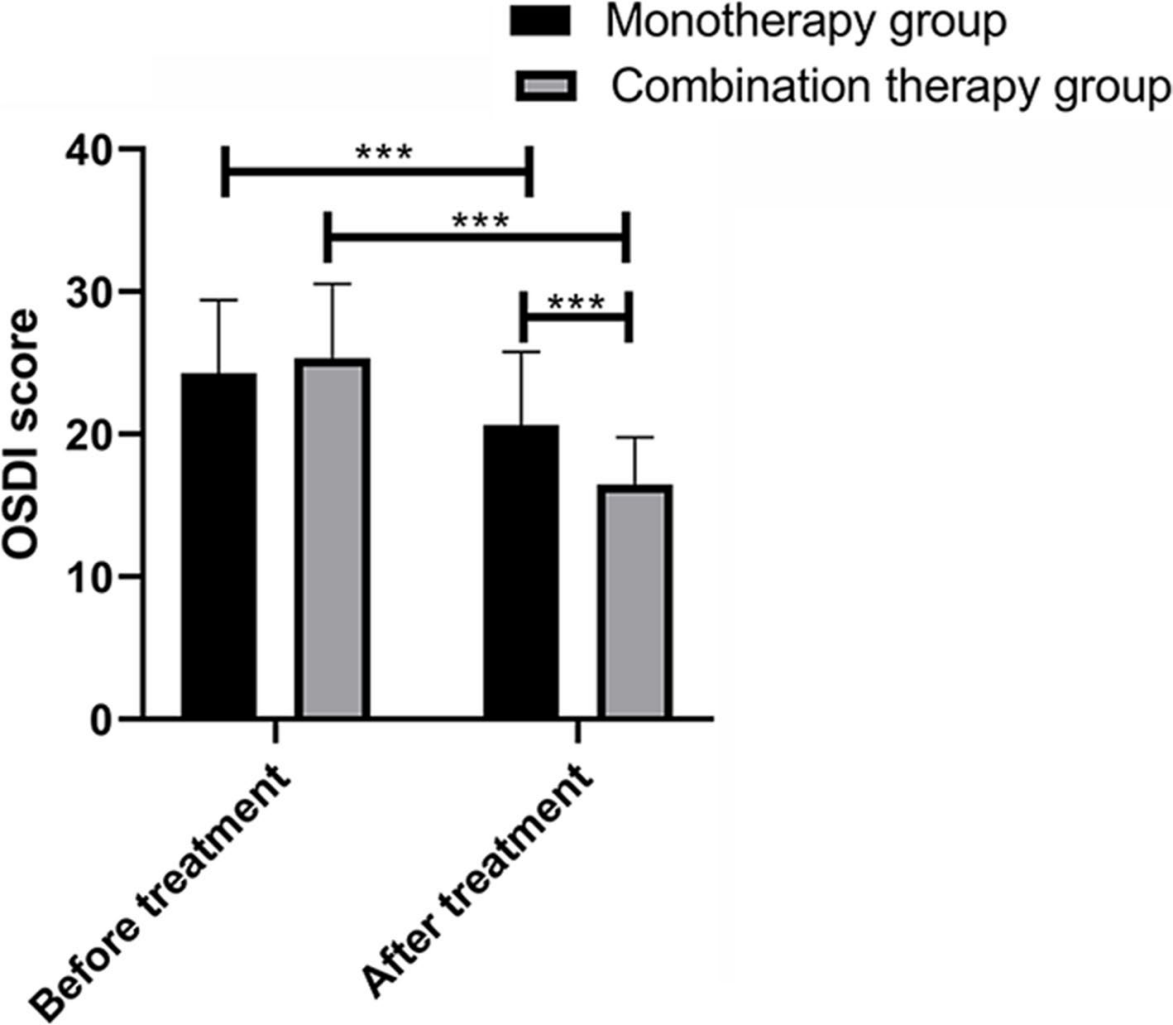
There was no significant difference in OSDI score between the 2 groups before treatment. After treatment, the OSDI scores of both groups decreased (*P* < 0.001), and the OSDI score of the combination group was significantly lower than that of the monotherapy group (*P* < 0.001). *** indicates *P* < 0.001

Comparison of OSDI score between the 2 groups before and after treatment revealed that there was no significant difference in OSDI score between the two groups before treatment (*P* > 0.05). After treatment, the OSDI scores of both groups decreased (*P* < 0.001), and the CG showed reduced OSDI score compared with the MG (*P* < 0.001) (Fig. 1).

### Comparison of BUT, FL, and SI levels

After treatment, the BUT and SI levels of both groups increased significantly (both *P* < 0.001), and the CG showed increased BUT and SI levels compared with the MG (both *P* < 0.001). In addition, after treatment, the FL levels of both groups decreased (both *P* < 0.001), and the FL level of the CG was significantly lower than that of the control group (*P* < 0.001) (Table 3).

**Table 3:** Comparison of BUT, FL, and SI values between the 2 groups before and after treatment

Group	BUT, s		FL, points		SI, mm	
	Before treatment	After treatment	Before treatment	After treatment	Before treatment	After treatment
Monotherapy group (*n* = 50)	6.22 ± 2.12	10.37 ± 3.18*	2.58 ± 0.17	1.04 ± 0.18*	6.63 ± 2.11	11.53 ± 3.05*
Combination group (*n* = 56)	6.17 ± 1.95	13.42 ± 3.22*	2.61 ± 0.21	0.45 ± 0.12*	6.57 ± 1.94	14.42 ± 3.26*
*t*	0.127	7.806	0.802	20.05	0.153	
*P* value	0.899	< 0.001	0.424	< 0.001	0.879	< 0.001

*P < 0.05 compared with those before treatment

### Comparison of clinical efficacy

According to statistics of the clinical efficacy on the 2 groups, the total effective rate of the CG was significantly higher than that of the MG (92.86% vs 78.00%) (Table 4).

**Table 4:** Comparison of clinical efficacy

Group	No. of cured patients	Patients with marked effective treatment	Patients with effective treatment	Patients without effective treatment	Patients with markedly effective or effective treatment
Monotherapy group (*n* = 50)	11 (22.00)	16 (32.00)	12 (24.00)	11 (22.00)	38 (78.00)
Combination group (*n* = 56)	19 (33.93)	22 (39.29)	11 (19.64)	4 (7.14)	52 (92.86)
χ^2^					5.000
*P* value					0.025

### Comparison of IL-1, IL-6, and TNF-α levels

Before treatment, there was no significant difference between the 2 groups in the levels of IL-1, IL-6, and TNF-α (all *P* > 0.05). After treatment, the levels of IL-1, IL-6, and TNF-α in both groups decreased (all *P* < 0.001). The levels in the CG were significantly lower than those in the MG (all *P* < 0.001) (Table 5).

**Table 5:** Comparison of IL-1, IL-6, and TNF-α levels between the 2 groups before and after treatment

Group	IL-1 (ng/mL)		IL-6 (ng/mL)		TNF-α (ng/mL)	
	Before treatment	After treatment	Before treatment	After treatment	Before treatment	After treatment
Monotherapy group (*n* = 50)	47.28 ± 8.25	31.45 ± 5.08*	45.37 ± 6.38	29.73 ± 5.04*	53.26 ± 9.06	33.79 ± 6.58*
Combination group (*n* = 56)	49.53 ± 8.32	23.74 ± 5.21*	44.56 ± 6.12	20.55 ± 4.82*	54.57 ± 8.85	25.38 ± 6.14*
*t*	1.395	7.696	0.667	9.580	0.752	6.806
*P* value	0.166	< 0.001	0.506	< 0.001	0.454	< 0.001

*P < 0.05 compared with those before treatment

### Correlation of IL-1, IL-6, and TNF-α with clinical efficacy

**Fig. 2:**
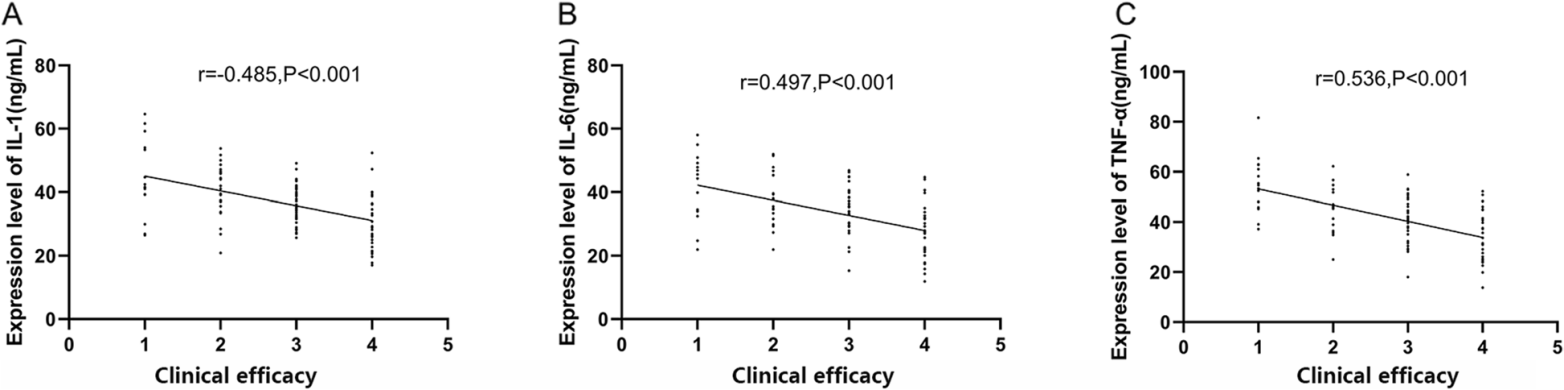
Correlation of IL-1, IL-6, and TNF-α with clinical efficacy. **A** With the increase of IL-1 level, the clinical efficacy gradually declined, and IL-1 level was negatively correlated with clinical efficacy (*r* = 0.485, *P* < 0.001). **B** With the increase of IL-6 level, the clinical efficacy gradually declined, and IL-6 level was negatively correlated with clinical efficacy (*r* = 0.497, *P* < 0.001). **C** With the increase of TNF-α level, the clinical efficacy gradually declined, and TNF-α level was negatively correlated with clinical efficacy (*r* = 0.536, *P* < 0.001)

In order to explore the correlation of IL-1, IL-6, and TNF-α with clinical efficacy, we grouped all patients according to clinical efficacy; collected the levels of serum IL-1, IL-6, and TNF-α in each group before treatment; and analyzed the correlation of IL-1, IL-6, and TNF-α with clinical efficacy using Spearman correlation analysis. We observed that the clinical efficacy gradually declined with an increase in IL-1, IL-6, and TNF-α, and that IL-1, IL-6, and TNF-α were negatively correlated with clinical efficacy (*r* = 0.485 and *P* < 0.001; *r* = 0.497 and *P* < 0.001; and *r* = 0.536 and *P* < 0.001) (Fig. 2).

### Predictive value of IL-1, IL-6, and TNF-α in Xerophthalmia

**Fig. 3:**
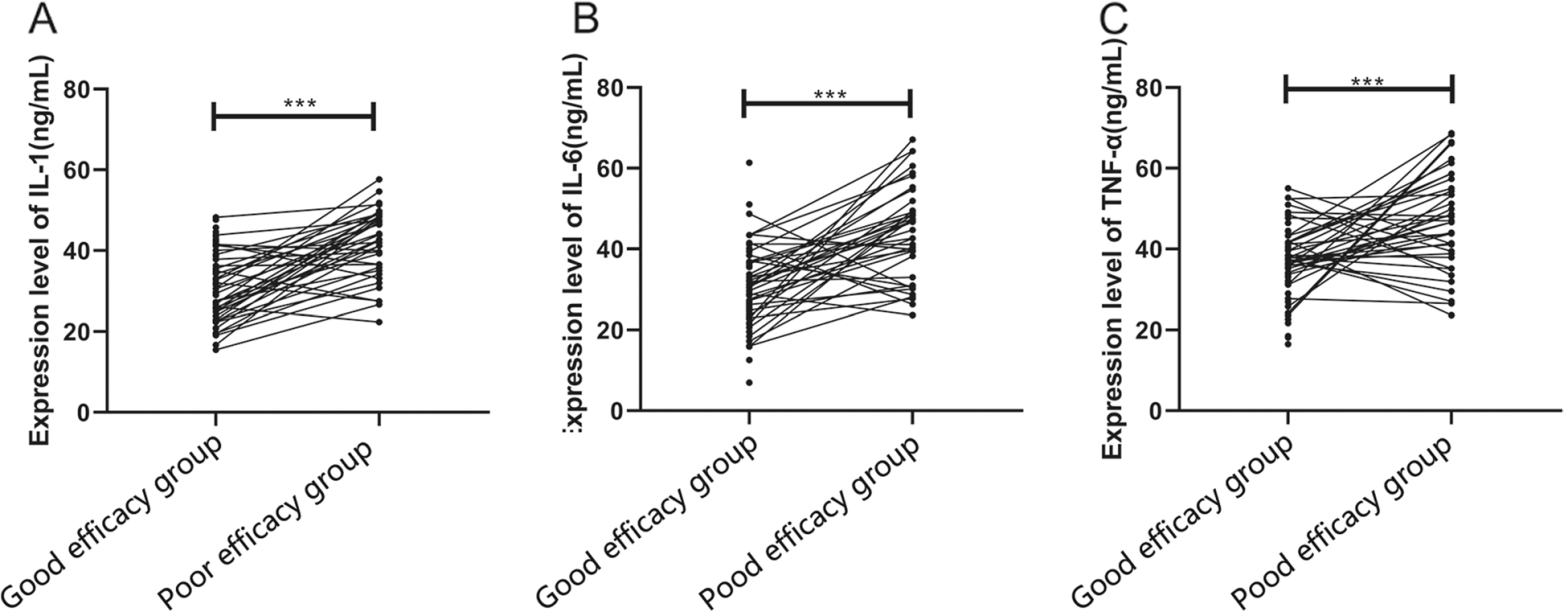
**A** The level of IL-1 in the good efficacy group was significantly lower than that in the poor efficacy group. **B** The level of IL-6 in the good efficacy group was significantly lower than that in the poor efficacy group. C The level of TNF-α in the good efficacy group was significantly lower than that in the poor efficacy group. * * * indicates *P* < 0.001

**Fig. 4:**
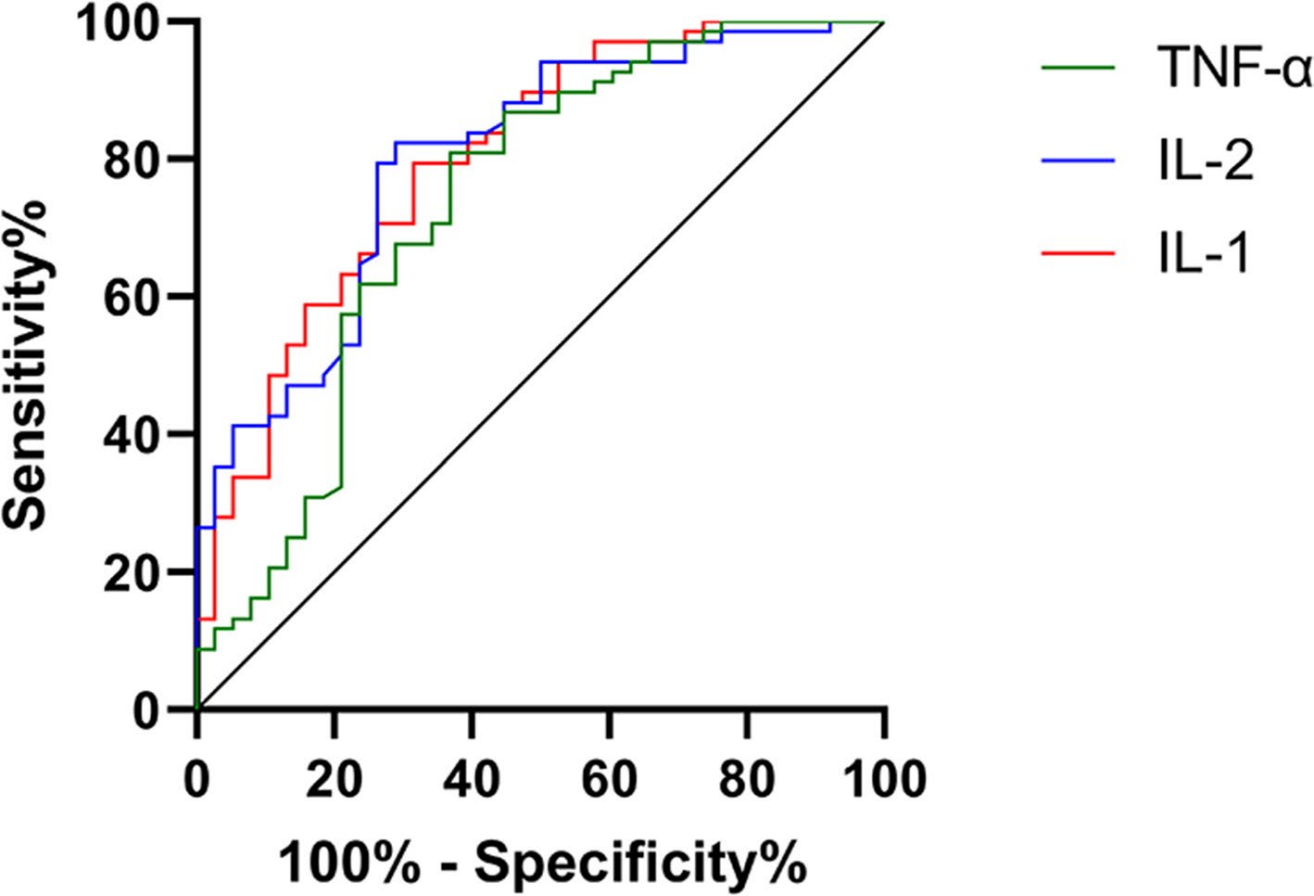
ROC curves showing the predictive value of IL-1, IL-6, and TNF-α in the efficacy on xerophthalmia. The red line indicates the ROC curve of the predictive value of IL-1 in the efficacy on xerophthalmia, and the area under the curve was 0.801. The blue line indicates the ROC curve of the predictive value of IL-6 in the efficacy on xerophthalmia, and the area under the curve was 0.800. The green line indicates the ROC curve of the predictive value of TNF-α in the efficacy on xerophthalmia, and the area under the curve was 0.736

To explore the predictive value of IL-1, IL-6, and TNF-α in the efficacy on xerophthalmia, we divided the patients into 2 groups according to clinical efficacy: a good efficacy group (*n* = 68 [cured patients + patients with markedly effective treatment]) and a poor efficacy group (*n* = 38 [patients with effective treatment + patients with ineffective treatment]). We then compared the levels of IL-1, IL-6, and TNF-α between the 2 groups before treatment. It was found that the levels of IL-1, IL-6, and TNF-α in the good efficacy group were significantly lower than those in the poor efficacy group before treatment (all *P* < 0.001) (Fig. 3). The ROC curves showed that the areas under the curves of IL-1, IL-6, and TNF-α were 0.801, 0.800, and 0.736, respectively, which indicated that IL-1, IL-6, and TNF-α had a high predictive value in efficacy (Fig. 4).

## Discussion

Xerophthalmia is an ocular surface disease caused by various factors, which results in instability of tear film, increase of osmotic pressure, ocular surface inflammation and injury, and nerve sensory abnormalities, and it is mainly manifested as dry eye, itching, foreign body sensation, and blurred vision [21]. Cataract surgery may result in slight ocular surface damage and xerophthalmia, and may even aggravate previous ocular surface diseases, which directly leads to a patients’ dissatisfaction with the effect of the cataract surgery [22]. Xerophthalmia has become a common problem after ophthalmic surgery, and with the increase of age, the symptoms of postoperative xerophthalmia deteriorate significantly, affecting the effectiveness of the operation and the quality of life of patients [23]. Therefore, it is particularly important to treat postoperative xerophthalmia.

The pathogenesis of xerophthalmia is complicated. Immune regulation is considered as the core mechanism of xerophthalmia, and anti-inflammation is the key to treat this disease. The OSDI questionnaire is one of the most widely used questionnaires to evaluate the subjective symptoms of xerophthalmia, which is closely related to the severity of diseases [24]. We first evaluated the OSDI scores of the 2 groups and found that, after treatment, the OSDI scores of both groups decreased and that the OSDI score of the CG was significantly lower than that of the MG, indicating that combined therapy was more effective in reliving the symptoms of xerophthalmia and reducing the severity of ocular surface diseases. Patients with xerophthalmia experience strong discomfort around the eyes and their visual function may be compromised owing to xerophthalmia [25]. BUT, FL, and SI are commonly used indicators for evaluation of the ocular function of patients with xerophthalmia [26–28]. Patients with xerophthalmia have corneal epithelial damage accompanied by tear secretion dysfunction and relatively short BUT [29, 30]. Therefore, we evaluated the BUT, FL, and SI of the 2 groups and found that that the CG demonstrated higher BUT and SI levels and a lower FL level, indicating that combined therapy was more effective in improving the ocular function of patients. Sodium hyaluronate is an ideal artificial tear, with good biocompatibility. With its good moisturizing and lubricating functions, sodium hyaluronate can effectively relieve symptoms of xerophthalmia and accelerate the repair and healing of corneal epithelial damage [31]. One study by Szegedi et al. [32] revealed that eye drops containing sodium hyaluronate can alleviate the symptoms of xerophthalmia, improve BUT, FL, and SI levels of patients with xerophthalmia, and increase the thickness of the lipid layer. However, the effect of sodium hyaluronate alone on the alleviation of xerophthalmia symptoms and visual function is not as significant as that of combined therapy [33]. According to some studies, epidermal growth factor has demonstrated good effect and high safety in the treatment of dermatitis, wound healing, oropharyngeal and upper esophageal mucosal diseases, and some corneal or conjunctival mucosal lesions [34]. In addition, epidermal growth factor eyedrops can ameliorate keratopathy after refractive surgery and diabetic keratopathy [35]. We evaluated the clinical efficacy on the 2 groups and found that the total effective rate of the CG was significantly higher than that of the MG, indicating that combined therapy could significantly ameliorate the symptoms of xerophthalmia after cataract surgery and increase the effectiveness of the treatment. Thus, it was found to be conducive to improving the satisfaction of patients undergoing cataract surgery. The better therapeutic effect of sodium hyaluronate combined with rhEGF may be owing to the fact that sodium hyaluronate relieves the symptoms of xerophthalmia, whereas rhEGF accelerates the amelioration of clinical symptoms by promoting the differentiation and proliferation of corneal epithelial cells, thus greatly shortening the healing time and ameliorating the symptoms of xerophthalmia when used in combination.

Immune regulation and inflammatory response are crucial in the pathogenesis of xerophthalmia. Inflammatory factors released during corneal incision healing will also lead to instability of tear film, thereby reducing corneal sensitivity [36]. Studies by Baudouin et al. [37] and Buzzi et al. [38] reported that the levels of IL-1, IL-6, and TNF-α in patients with ocular surface diseases are high and that the high levels interfere with ocular surface homeostasis. Therefore, we quantified the levels of inflammatory factors in the 2 groups, finding that the levels of IL-1, IL-6, and TNF-α in the CG were significantly lower than those in the MG, which indicated that combined therapy could significantly relieve inflammation and promote the amelioration of xerophthalmia. A study by Simmons et al. [39] revealed that the cornea and conjunctiva of mice produce significantly fewer inflammatory factors after knockout of IL-1 receptor in the mice. Another study by Yang et al. [40] demonstrated that Mingmu Yanggan pills combined with sodium hyaluronate eyedrops can lower the levels of IL-33 and IL-6 in the tears of perimenopausal women with xerophthalmia. We analyzed the correlation between clinical efficacy and the levels of inflammatory factors through Spearman correlation analysis and found that clinical efficacy gradually declined with the increase of IL-1, IL-6, and TNF-α, which indicated that IL-1, IL-6, and TNF-α can be used as potential outcome measures for clinical efficacy on patients with xerophthalmia. Therefore, we divided the patients into 2 groups, a good efficacy group and a poor efficacy group, according to clinical efficacy, and collected the statistics of IL-1, IL-6, and TNF-α levels in both groups before treatment. We found that the levels in the good efficacy group were significantly lower than those in the other group. We also drew ROC curves of the predictive value of IL-1, IL-6, and TNF-α in efficacy. The areas under the curves of IL-1, IL-6, and TNF-α were 0.801, 0.800 and 0.736, respectively, indicating that IL-1, IL-6, and TNF-α in tears of patients with xerophthalmia have a high predictive value in efficacy.

In previous studies, cataract surgery and postoperative used eye drops that trigger Meibomian gland dysfunction [41–43], because researchers considered that the cataract surgery and postoperative used eye drops may make meibum easy to discharge. However, in this study, patients who underwent Sodium Hyaluronate Combined with rhEGF treatment did not undergo Meibomian gland dysfunction because there were no high-restorative patients enrolled in this study. This study has confirmed that sodium hyaluronate combined with rhEGF is helpful to relieve clinical symptoms and inflammation in patients with xerophthalmia after cataract surgery. However, another study has concluded that different dosages of sodium hyaluronate have different therapeutic effects [44]. The drug dosage used in this study was the same for all patients, so we do not know whether the dosage adopted in this study has the best therapeutic effect. Therefore, we hope to increase drug dosages in future studies to further explore the efficacy of combined therapy.

## Conclusions

In conclusion, sodium hyaluronate combined with rhEGF is helpful to ameliorate clinical symptoms and inflammation of patients with xerophthalmia after cataract surgery.

## Data Availability

The data used to support the findings of this study are available from the corresponding author upon request.

## Abbreviations

rhEGF: Recombinant human epidermal growth factor
MG: Monotherapy group
CG: Combination group
OSDI: Ocular surface disease index
BUT: Break-up time
SI: Schirmer I test
IL: Interleukin
TNF-α: tumor necrosis factor-α
ELISA: Enzyme-linked immuno-sorbent assay
FL: Fluorescein corneal staining
ROC: Receiver operating characteristic
